# A Novel Mutation in *FOXC1* in a Lebanese Family with Congenital Heart Disease and Anterior Segment Dysgenesis: Potential Roles for *NFATC1* and *DPT* in the Phenotypic Variations

**DOI:** 10.3389/fcvm.2017.00058

**Published:** 2017-09-20

**Authors:** Athar Khalil, Christiane Al-Haddad, Hadla Hariri, Kamel Shibbani, Fadi Bitar, Mazen Kurban, Georges Nemer, Mariam Arabi

**Affiliations:** ^1^Department of Biochemistry and Molecular Genetics, American University of Beirut, Beirut, Lebanon; ^2^Department of Ophthalmology, American University of Beirut, Beirut, Lebanon; ^3^Department of Pediatrics and Adolescent Medicine, American University of Beirut, Beirut, Lebanon; ^4^Department of Dermatology, American University of Beirut, Beirut, Lebanon; ^5^Department of Dermatology, Columbia University, New York, NY, United States

**Keywords:** anterior segment dysgenesis, congenital heart disease, forkhead box c1, digenic, whole exome sequencing

## Abstract

Congenital heart diseases (CHDs) are still the leading cause of death in neonates. Anterior segment dysgenesis is a broad clinical phenotype that affects the normal development of the eye, leading in most of the cases to glaucoma which is still a major cause of blindness for children and adolescents. Despite tremendous insights gained from genetic studies, a clear genotype–phenotype correlation is still difficult to draw. In Lebanon, a small country with still a high rate of consanguineous marriages, there are little data on the epidemiology of glaucoma amongst children with or without CHD. We carried out whole exome sequencing (WES) on a family with anterior segment dysgenesis, and CHD composed of three affected children with glaucoma, two of them with structural cardiac defects and three healthy siblings. The results unravel a novel mutation in *FOXC1* (p. R127H) segregating with the phenotype and inherited from the mother, who did not develop glaucoma. We propose a digenic model for glaucoma in this family by combining the *FOXC1* variant with a missense variant inherited from the father in the dermatopontin (*DPT*) gene. We also unravel a novel *NFATC1* missense mutation predicted to be deleterious and present only in the patient with a severe ocular and cardiac phenotype. This is the first report on *FOXC1* using WES to genetically characterize a family with both ocular and cardiac malformations. Our results support the usage of such technology to have a better genotype–phenotype picture for Mendelian-inherited diseases for which expressivity and penetrance are still not answered.

## Introduction

Glaucoma is the second leading cause of blindness in the world, according to the World Health Organization (1, 2). It is characterized by a progressive damage of the eye’s optic nerve, which is highly influenced by fluid building up in the front part of the eye leading to an increase in the intraocular pressure (IOP) (3, 4). Genetic predisposition, aging, and environmental factors do play essential roles in the developing of the disease in adults; however, the financial, social, and familial burden is more sensed in the congenital and juvenile cases (3, 5–8). These cases are mostly linked to developmental defects in the formation and differentiation of the cells that make the ocular system in general, and in particular the optic nerve and the anterior segment which comprise the iris, the lens, and the cornea (3, 5, 6).

Pediatric glaucomas are divided into two major types: primary congenital glaucoma (PCG), which accounts for 50–70% of all childhood cases, and syndromic-associated glaucomas that include among others juvenile open angle glaucoma (JOAG) (OMIM#37750, #603383, and #137760), aniridia (OMIM#106210), and the Axenfeld–Rieger syndrome (ARS) (OMIM#180500, #601499, and #602482). Linkage analyses and genome wide association studies have identified many loci and genes implicated in PCG, mostly inherited in an autosomal recessive form, diagnosed in the first year of life, and largely prevalent in countries with high consanguinity. Among these, *MYOC* (myocilin) and *CYP1B1* (cytochrome P450) represent the frequently mutated genes linked to the phenotype (6, 9). Interestingly, a digenic mode of inheritance with both *CYP1B1* and *MYOC* mutations has been documented in patients with PCG highlighting the potential involvement of common genetic and molecular players in the disease (10). JOAG is inherited mainly as a dominant trait with an onset age ranging from 3 to 35 years and characterized by high IOP requiring in most of the cases early surgical treatment. The major genetic players are mutations in *MYOC* with high prevalence and penetrance, followed by *OPTN* (optineurin) and *WDR36* (WD repeat containing protein 36) (11). Aniridia is a very rare panocular disease whereby glaucoma is diagnosed in 50–70% of the cases at later ages (end of adolescent, early adulthood). Mostly inherited as autosomal dominant, it is mainly caused by mutations in *PAX6* (paired-box gene 6), the master regulator gene of eye development (12, 13). As for ARS, it is an autosomal dominant disorder characterized by iris stromal hypoplasia, prominent Schwalbe line (embryotoxon), adhesion between the iris and Schwalbe line, microcornea, corneal opacity, and increased IOP that leads to glaucoma in about 50% of the cases (3, 14–17). Patients with ARS can also have maxillary hypoplasia, dental anomalies, umbilical hernia, and/or hypospadias. More rarely, they may have hydrocephalus, hearing loss, cardiac and kidney abnormalities, and congenital hip dislocation in addition to the ocular abnormalities. Globally, mutations in two genes *FOXC1* (forkhead box c1) and *PITX2* (paired-like homeodomain transcription factor 2) have been shown to be responsible for most of the cases with complete penetrance but variable expressivity (14, 17–19).

FOXC1 belongs to the Forkhead box (FOX) family of transcription factors, which share an evolutionarily conserved DNA-binding domain known as the Forkhead domain (20–22). In addition to the eye, it plays a dose-dependent evolutionary conserved role in the early development of the blood vessels, the brain, the heart, and the somites (23). *Foxc1* homozygous null mutant mice have a lethal phenotype; they die pre- and perinatally with hemorrhagic hydrocephalus and multiple skeletal, ocular, and genitourinary defects. They also suffer from cardiovascular defects, most notably, interruption or coarctation of the aortic arch (24, 25). In addition to its critical role during ocular development, *Foxc1* has a protective role in the adult eye. It regulates antiapoptotic genes such as *Foxo1a* in order to maintain homeostasis in the adult trabecular meshwork (TM), which is constantly exposed to aqueous humor (26). By maintaining programmed cell death and thus proliferation of TM cells, *FOXC1* is a major player in the onset of glaucoma once deregulated. In humans, deleterious *FOXC1* mutations associated with ARS span the entire region of the protein including the DNA-binding and activation domains, resulting in reduced transcriptional activity due to haploinsufficiency. Despite the *in vitro* studies that assess these differential activities, a genotype–phenotype correlation map could not be established to explain the variable expressivity of the phenotype even among patients harboring the same mutation.

In Lebanon, a small country with still frequent consanguineous marriages, only one recent study addressed the genetic basis of congenital glaucoma (27). We hereby describe the first *FOXC1* missense mutation using whole exome sequencing (WES) in a Lebanese family with anterior segment dysgenesis and cardiac phenotypes. We showed that this novel mutation has different expressivity, but we propose a digenic model of inheritance that includes *NFATC1* to explain the severe cardiac phenotype in one of the patients. Our findings explicitly call for a revision of the expressivity and penetrance terms in genetic inheritance by using WES as a tool to explain genotype–phenotype correlations.

## Materials and Methods

### Patients Recruitment

The study was approved by the institutional review board at the American University of Beirut. All patients, their legal guardians, and family members signed an informed consent form before being enrolled in the study. Patients presenting to the Department of Pediatrics and Adolescent Medicine with Congenital Heart Disease (CHD) were serially recruited in the study under IRB approved protocol BioCh.Gn.01. Blood samples were collected from a family consisting of six children and their parents. Two children presented with glaucoma and VSD and one with glaucoma only. Standard clinical evaluation included a complete physical examination, electrocardiography and two-dimensional transthoracic echocardiography with color Doppler were obtained. Family consanguinity history was utilized in constructing pedigrees after interviewing all patients and their parents.

### Exome Sequencing

Blood samples were collected from all members, and the DNA was extracted using the Qiagen Blood-Midi kit (Qiagen Science Inc., Germantown, MD, USA), following the manufacturer’s protocol. DNA quantification was performed using the NanoDrop (Thermo Fisher Scientific Inc., Waltham, MA, USA) at the molecular core facility at AUB. One microgram of coded DNA samples from both parents, and the six children were shipped to Macrogen (South Korea, www.dna.macrogen.com) where exome sequencing was performed using the V6 Sureselect target enrichment capture system from Agilent on a HiSeq4000 platform from Illumina (San Diego, CA, USA).

### Sanger Sequencing

Sanger sequencing was used to confirm the missense mutation in *FOXC1* by exome sequencing. Briefly, primers were designed to amplify partially the region on exon 1 of the gene that covers the mutation: 5′-CCTACGGGCCCTACACG-3′ (F) and 5′-GTTGTCCACGCTGAAGCC-3′ (R). The 749 bp PCR products were resolved on agarose gels, then purified using the QIAquick kits (Qiagen, Science Inc. Germantown, MD, USA). Sanger sequencing was carried out as previously described on an ABI3500 (Applied Biosystems, Foster City, CA, USA) platform at the American University of Beirut Molecular Core Facility.

### Data Analysis

Primary analysis was done at Macrogen. The Fastq files were mapped to the reference genome using the Burrows–Wheeler Alignment tool. The Genome Analysis Toolkit was used for variants calling, and the SnEff software was used to annotate the variants. The Illumina Variant Studio was used to filter the variants as per their frequency and presence or absence in the affected family members versus the healthy individuals.

## Results

### Clinical Evaluation: Is Glaucoma Separate from CHD?

**Figure 1:**
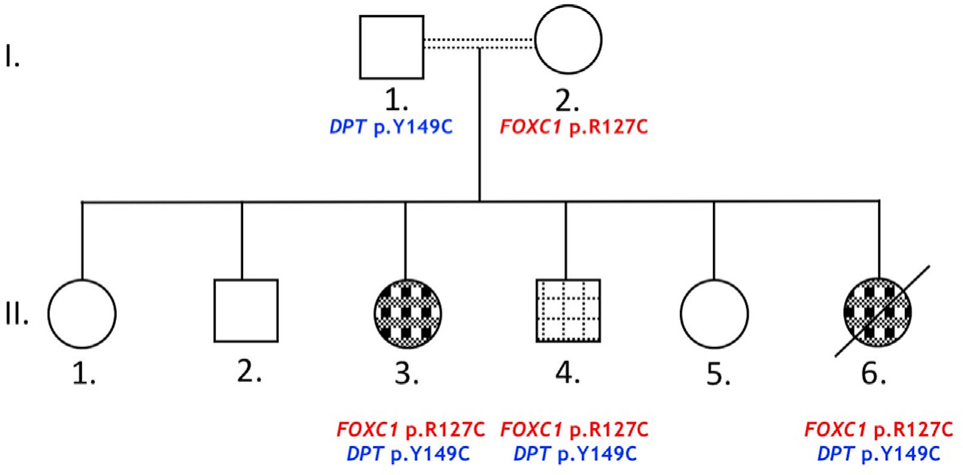
Glaucoma and congenital heart disease in a Lebanese family. The pedigree shows a two-generations family (roman numbers): circles and squares for females and males, respectively. Primary glaucoma and aniridia (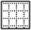) or glaucoma, aniridia, and congenital heart defects (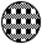). Double lines indicate first degree cousin marriages. Death is represented with an oblique black line.

Index patient II.3 (Figure 1) was referred to the Pediatrics Heart Center at the American University of Beirut Medical Center for consultation at the age of 8 years. Echocardiography showed a small perimembranous ventricular septal defect (VSD) with aneurysmal tissue formation. The color Doppler study was indicative of normal pulmonary arterial pressure with no valvar stenosis. There was no need for cardiac medications, and the parents were advised to a follow-up visit after 2 years. Concomitant with this date, the parents had a newborn girl, patient II.6 who was admitted to the emergency room for congestive heart failure at 4 months of age. Echocardiography results showed a mild biventricular hypertrophy, with a more prominent right ventricle phenotype. A large subaortic VSD was noted with an overriding aorta, and pulmonary atresia reminiscent of a Tetralogy of Fallot (TOF)-like phenotype. The color Doppler imaging showed a trace tricuspid regurgitation, and a patent ductus arteriosus (PDA) like structure arising most likely from the transverse aorta. A right-modified Blalock–Taussig shunt was performed, and in parallel the patient was diagnosed with aniridia and glaucoma. Her ocular examination at the age of 6 months showed partial aniridia in both eyes with corneal edema on the right and corneal opacification with neovascularization on the left. Examination under anesthesia revealed elevated IOP at 21 mmHg with enlarged corneas measuring 11 mm on the right and 13 mm on the left. Dilated fundoscopy showed a normal posterior pole on the right with a cup to disk ratio of 0.4. No view could be obtained of the left fundus. She underwent partial trabeculotomy in the right eye as it was as discovered intraoperatively that she had incomplete development of her Schlemm’s canal. This finding prohibited complete trabeculotomy and confirmed an anterior segment dysgenesis diagnosis. Two months later, she underwent Ahmed tube implantation in the right eye due to persistent glaucoma. Her postoperative course was complicated by a right corneal ulcer and an infection of the shunt that necessitated its removal 3 months after placement. She subsequently did well postoperatively with a limited hyphema and was lost to follow-up later. The same ocular finding was also detected in two of her siblings (II.3 and II.4), but was absent from the remaining four. The parents were not examined at our facilities, but they denied having any cardiac or ocular problem. The family was enrolled in our ongoing genetic study on CHDs, but patient II.6 passed away few months later, and no follow-up was made with the rest of the family members.

### Genetic Analysis (I): A Novel FOXC1 Missense Variant with Variable Expressivity

**Figure 2:**
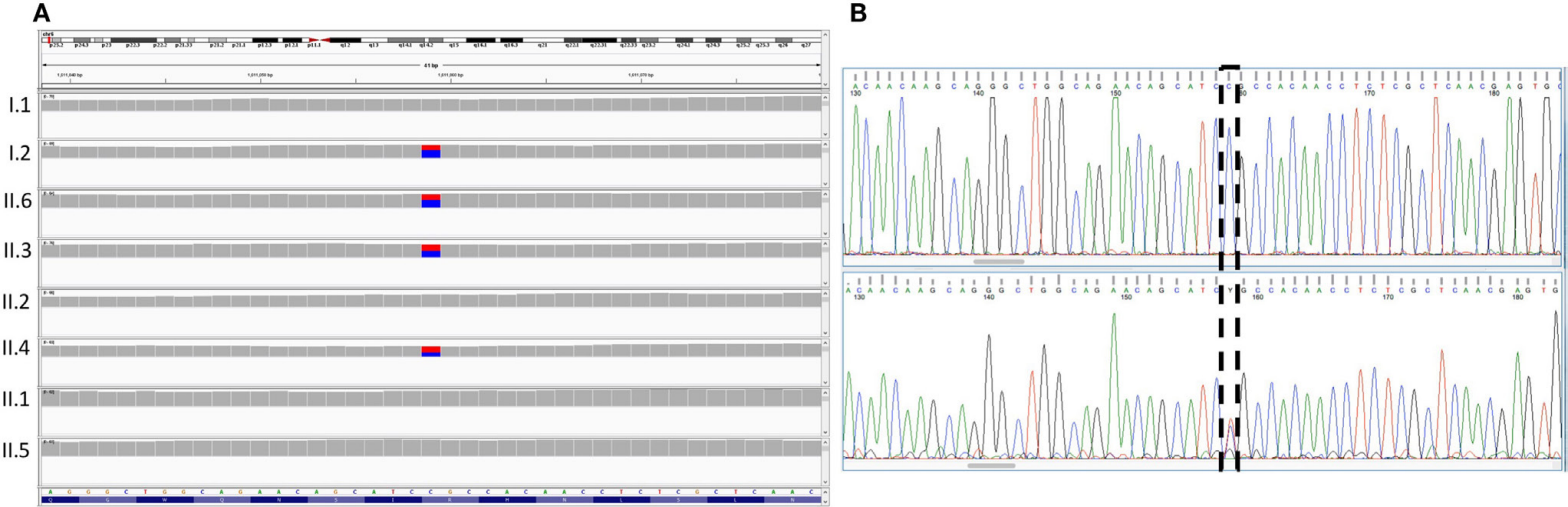
Sequencing results for the *FOXC1* variant p.R127C. **(A)** Integrative genomics viewer visualization of whole exome sequencing shows a novel heterozygous variant (blue for the normal “C” allele and red for the variant “T” allele) in *FOXC1* gene in family members I.2, II.3, II.2, and II.6. **(B)** Sanger sequencing of the *FOXC1* gene confirmed the C>T variant (boxed), in the affected individuals represented in the lower panel versus the normal individuals in the upper panel.

The results of WES yielded an average of 98,521 variants enclosing both single nucleotide polymorphisms and insertions/deletions in the six children and their parents. A first round of variants’ filtering consists of keeping only variants from an inclusive list of 65 genes (Table S1 in Supplementary Material) implicated in glaucoma, anterior segment dysgenesis, microcornea, and microphthalmia. We use an arbitrary model of inheritance that includes X-linked recessive, or autosomal dominant, or autosomal recessive with a minor allele frequency (MAF) less than 5%, and excluding synonymous and in-frame insertions/deletions variants. Only one novel variant in the coding region of *FOXC1* was detected in the three affected children (Table 1); the chr 6:1611059C>T variant leads to a missense mutation p.R127C (NM_001453.2) in the DNA-binding domain of the protein. While being absent from the father and the three non-affected children, this missense mutation is inherited from the healthy mother and is predicted to be deleterious and damaging as ascertained by Sanger sequencing (Figure 2). Additionally, a missense variant in *PCMTD1* was encountered in all family members, affected and unaffected, therefore excluding it from being responsible for the ocular phenotype (Table 1).

**Table 1:** Variants in genes implicated in glaucoma, anterior segment dysgenesis, microcornea, and microphthalmia in the affected patients.

Sample	Gene	Variant	Coordinate	Chr	Type	Filters	Quality	Inherited from	Allelic depths	Transcript	Consequence	Protein position	Amino acids	Sift	PolyPhen	dbSNP ID	Allele freq global minor	Allele Freq Evs	EVS Coverage	EVS Samples
II.6	CNTNAP2	C>C/CTG	148106477	7	Insertion	PASS	658.77	Father	21,18	NM_014141.5	splice_region_variant, intron_variant, feature_elongation	0					0	0	0	0

II.4	CNTNAP2	G>G/T	146805232	7	Snv	PASS	36.77	None	2,3	NM_014141.5	splice_region_variant, intron_variant	0					0	0	54	6503

II.6	COL11A1	GA>G/G	103496805	1	Deletion	PASS	78.03	Both	0,4	NM_080629.2	splice_region_variant, intron_variant, feature_truncation	0				rs67059272, rs36076089	0	0	20	6498

II.3	COL11A1	GA>G/G	103496805	1	Deletion	PASS	52.28	Both	0,3	NM_080629.2	splice_region_variant, intron_variant, feature_truncation	0				rs67059272, rs36076089	0	0	20	6498

II.4	COL11A1	GA>G/G	103496805	1	Deletion	PASS	52.28	Both	0,3	NM_080629.2	splice_region_variant, intron_variant, feature_truncation	0				rs67059272, rs36076089	0	0	20	6498

II.6	FOXC1	C>C/T	1611059	6	Snv	PASS	615.77	Mother	28,20	NM_001453.2	missense_variant	127	R/C	deleterious(0)	probably_damaging(1)		0	0	96	6503

II.3	FOXC1	C>C/T	1611059	6	Snv	PASS	896.77	Mother	27,28	NM_001453.2	missense_variant	127	R/C	deleterious(0)	probably_damaging(1)		0	0	96	6503

II.4	FOXC1	C>C/T	1611059	6	Snv	PASS	704.77	Mother	15,22	NM_001453.2	missense_variant	127	R/C	deleterious(0)	probably_damaging(1)		0	0	96	6503

II.6	GALC	GA>G/G	88417095	14	Deletion	PASS	282.1	Both	1,12	NM_000153.3	splice_region_variant, intron_variant, feature_truncation	0				rs11300320	0	0	11	5784

II.3	GALC	GA>G/G	88417095	14	Deletion	PASS	799.77	Both	0,30	NM_000153.3	splice_region_variant, intron_variant, feature_truncation	0				rs11300320	0	0	11	5784

II.4	GALC	GA>G/G	88417095	14	Deletion	PASS	477.77	Both	0,18	NM_000153.3	splice_region_variant, intron_variant, feature_truncation	0				rs11300320	0	0	11	5784

II.6	PCMTD1	A>A/C	52733079	8	Snv	PASS	76.77	None	20,4	NM_052937.2	missense_variant	302	S/R	tolerated(0.09)	probably_damaging(0.975)	rs75865149	0	0	115	6503

II.6	PCMTD1	T>T/G	52733128	8	Snv	PASS	238.77	Ambiguous	15,9	NM_052937.2	missense_variant	286	N/T	tolerated(0.56)	benign(0.012)	rs62506083	0	0	188	6503

II.3	PCMTD1	T>T/G	52733128	8	Snv	PASS	81.77	Ambiguous	14,5	NM_052937.2	missense_variant	286	N/T	tolerated(0.56)	benign(0.012)	rs62506083	0	0	188	6503

II.4	PCMTD1	T>T/G	52733128	8	Snv	PASS	187.77	Ambiguous	19,11	NM_052937.2	missense_variant	286	N/T	tolerated(0.56)	benign(0.012)	rs62506083	0	0	188	6503

### Genetic Analysis (II): A DPT Missense Variant Inherited from the Father Exclusively in the Affected Glaucomatous Patients

In order to understand the absence of ocular manifestations in the mother, we hypothesized that an additional variant inherited from the father with no effect on its own might be needed in conjunction with the *FOXC1* variant to cause Glaucoma in the affected children. Using the Illumina variant studio, we filtered the variants that are only shared by the three affected individuals (II.3, 4, and 6) and their father I.1 (Figure 1). The filtering process included all coding variants with an MAF < 5% and excluded synonymous and in-frame insertions/deletions variants. Among the six variants (Table 2), two are missense variants in the RNA Binding Motif Protein 43 (*RBM43*) gene (p.V34L) and the dermatopontin (*DPT*) gene (p.Y149C). The latter is novel, not reported previously in any database, and predicted to be deleterious and damaging (Table 2), while the former is reported in the SNP database, and the *RBM43* gene was not shown to be expressed in the eye. On the contrary, previous reports do support a role for *DPT* in the eye and potentially in glaucoma, thus reinforcing its potential role in the underlying phenotype especially that it is not expressed in the mother. The remaining four variants do not have an effect on the coding sequences or splicing, thus excluding them from any role in the ocular phenotype.

**Table 2:** Variants shared by affected patients inherited exclusively from the father.

Sample	Gene	Variant	Coordinate	Chr	Type	Filters	Quality	Inherited from	Allelic depths	Transcript	Consequence	Protein position	Amino acids	Sift	PolyPhen	dbSNP ID	Allele freq global minor	Allele freq EVS	EVS coverage	EVS samples
II.3	DPT	T>T/C	16870348	1	Snv	PASS	805.77	Father	34,28	NM_001937.4	missense_variant	149	Y/C	deleterious(0.04)	possibly_damaging(0.628)		0	0	172	6,503

II.4	DPT	T>T/C	16870348	1	Snv	PASS	997.77	Father	23,30	NM_001937.4	missense_variant	149	Y/C	deleterious(0.04)	possibly_damaging(0.628)		0	0	172	6,503

II.6	DPT	T>T/C	16870348	1	Snv	PASS	548.77	Father	28,21	NM_001937.4	missense_variant	149	Y/C	deleterious(0.04)	possibly_damaging(0.628)		0	0	172	6,503

II.3	INHBA	C>C/T	41729843	7	Snv	PASS	798.77	Father	28,27	NR_027118.1	upstream_gene_variant	0				rs138819536	0.09	0.26	51	6,503

II.4	INHBA	C>C/T	41729843	7	Snv	PASS	988.77	Father	29,33	NR_027118.1	upstream_gene_variant	0				rs138819536	0.09	0.26	51	6,503

II.6	INHBA	C>C/T	41729843	7	Snv	PASS	774.77	Father	21,27	NR_027118.1	upstream_gene_variant	0				rs138819536	0.09	0.26	51	6,503

II.3	POLR2J4, SPDYE1	G>G/A	44046965	7	Snv	PASS	1,115.77	Father	63,42	NR_003655.2	intron_variant, nc_transcript_variant	0				rs141407881	0.55	1.05	174	6,503

II.4	POLR2J4, SPDYE1	G>G/A	44046965	7	Snv	PASS	978.77	Father	54,37	NR_003655.2	intron_variant, nc_transcript_variant	0				rs141407881	0.55	1.05	174	6,503

II.6	POLR2J4, SPDYE1	G>G/A	44046965	7	Snv	PASS	966.77	Father	44,38	NR_003655.2	intron_variant, nc_transcript_variant	0				rs141407881	0.55	1.05	174	6,503

II.3	RBM43	C>C/G	152112161	2	Snv	PASS	907.77	Father	21,37	NM_198557.2	missense_variant	34	V/L	tolerated(0.8)	benign(0)	rs147060862	1.56	2.39	130	6,503

II.4	RBM43	C>C/G	152112161	2	Snv	PASS	680.77	Father	22,28	NM_198557.2	missense_variant	34	V/L	tolerated(0.8)	benign(0)	rs147060862	1.56	2.39	130	6,503

II.6	RBM43	C>C/G	152112161	2	Snv	PASS	278.77	Father	26,12	NM_198557.2	missense_variant	34	V/L	tolerated(0.8)	benign(0)	rs147060862	1.56	2.39	130	6,503

II.3	RTEL1, RTEL1-TNFRSF6B	G>G/A	62324290	20	Snv	PASS	1,215.77	Father	46,47	NM_003823.3	upstream_gene_variant	0				rs61736615	1.69	2.81	118	6,492

II.4	RTEL1, RTEL1-TNFRSF6B	G>G/A	62324290	20	Snv	PASS	941.77	Father	44,35	NM_003823.3	upstream_gene_variant	0				rs61736615	1.69	2.81	118	6,492

II.6	RTEL1, RTEL1-TNFRSF6B	G>G/A	62324290	20	Snv	PASS	1,239.77	Father	34,47	NM_003823.3	upstream_gene_variant	0				rs61736615	1.69	2.81	118	6,492

II.3	TIPRL	C>C/T	1.68E+08	1	Snv	PASS	1,016.77	Father	36,32	NM_152902.3	splice_region_variant, intron_variant	0					0	0.01	107	6,503

II.4	TIPRL	C>C/T	1.68E+08	1	Snv	PASS	573.77	Father	41,20	NM_152902.3	splice_region_variant, intron_variant	0					0	0.01	107	6,503

II.6	TIPRL	C>C/T	1.68E+08	1	Snv	PASS	893.77	Father	30,30	NM_152902.3	splice_region_variant, intron_variant	0					0	0.01	107	6,503

*EVS, Exome databases*.

### Genetic Analysis (III): A Novel Missense Variant in NFATC1 Responsible for the Severe Cardiac Phenotype?

To delineate the cardiac phenotype observed exclusively in probands II.3 and II.6, a variant filtering approach was conducted using a two-arm strategy. The variants should have the following characteristics; first, they must have an MAF < 5%, exclusively shared by the two individuals and absent from the others, and second, they must be inherited from both parents (assuming a recessive model of inheritance) or from the father assuming a combinatorial effect with the *FOXC1* variant. In the former strategy, only eight variants were shared (Table 3), but none could explain the cardiac phenotype alone or in conjunction with *FOXC1* since there are no published data about their role neither in cardiac development or in CHD. In the latter, 18 shared variants were detected with no cardiac relevance for anyone of them; of note a nonsense variant in *ZNF28* inherited from the father was not encountered in any database (Table 3). We moved then to assess each individual with cardiac defect alone by using the same strategy above. In patient II.3, 13 variants in a total of 7 genes were inherited from the father, however, none were previously implicated in CHD, nor were they implicated in a *FOXC1* partnership (Table 4). In contrast, patient II.6 had 17 variants in 15 genes inherited from her father but not encountered in the other siblings. Two of these variants were in genes implicated in heart development and cardiac pathology, *NFATC1* and *OBSCN* (Table 4). The two variants chr18:77170979 G>A for *NFATC1* and chr1:228462101 G>A for *OBSCN* lead to novel missense variants, p.R222Q and p.C1880Y respectively. They were not reported previously and are predicted to be deleterious and damaging, potentially explaining the severe cardiac phenotype in the patient (Table 4).

**Table 3:** Variants shared by patients with both glaucoma and congenital heart disease inherited either from the father alone or from both parents.

Sample	Gene	Variant	Coordinate	Chr	Type	Filters	Quality	Inherited from	Allelic depths	Transcript	Consequence	Protein position	Amino acids	Sift	PolyPhen	dbSNP ID	Allele freq global minor	Allele freq EVS	EVS coverage	EVS samples
II.3	CCDC155	C>C/G	49910139	19	Snv	PASS	816.77	Father	49,31	NM_144688.4	splice_region_variant, intron_variant	0				rs112074780	1.1	1.1	47	6,098

II.6	CCDC155	C>C/G	49910139	19	Snv	PASS	1,090.77	Father	54,39	NM_144688.4	splice_region_variant, intron_variant	0				rs112074780	1.1	1.1	47	6,098

II.3	CCDC9	T>T/C	47768055	19	Snv	PASS	529.77	Father	41,22	NM_015603.2	missense_variant	191	V/A	deleterious(0.01)	unknown(0)		0	0	19	6,496

II.6	CCDC9	T>T/C	47768055	19	Snv	PASS	630.77	Father	40,28	NM_015603.2	missense_variant	191	V/A	deleterious(0.01)	unknown(0)		0	0	19	6,496

II.3	DKKL1	G>G/A	49878275	19	Snv	PASS	1,317.77	Father	31,45	NM_014419.3	missense_variant	240	R/Q	tolerated(1)	benign(0.002)	rs35389403	0.73	0.54	25	6,444

II.6	DKKL1	G>G/A	49878275	19	Snv	PASS	882.77	Father	40,32	NM_014419.3	missense_variant	240	R/Q	tolerated(1)	benign(0.002)	rs35389403	0.73	0.54	25	6,444

II.3	KLK11	C>C/T	51527970	19	Snv	PASS	781.77	Father	51,34	NM_144947.1	missense_variant	73	E/K	tolerated(0.31)	benign(0.178)	rs117268623	1.74	1.94	26	6,503

II.6	KLK11	C>C/T	51527970	19	Snv	PASS	772.77	Father	34,31	NM_144947.1	missense_variant	73	E/K	tolerated(0.31)	benign(0.178)	rs117268623	1.74	1.94	26	6,503

II.3	LAMC2	G>G/A	183177132	1	Snv	PASS	808.77	Father	25,28	NM_005562.2	missense_variant	66	E/K	deleterious(0)	probably_damaging(0.995)	rs146325169	0	0.12	275	6,503

II.6	LAMC2	G>G/A	183177132	1	Snv	PASS	627.77	Father	21,27	NM_005562.2	missense_variant	66	E/K	deleterious(0)	probably_damaging(0.995)	rs146325169	0	0.12	275	6,503

II.3	OR8U1	C>C/A	56143976	11	Snv	PASS	61.77	Father	25,4	NM_001005204.1	missense_variant	293	Q/K	deleterious(0.03)	benign(0.003)		0	0	123	6,047

II.6	OR8U1	C>C/A	56143976	11	Snv	PASS	43.77	Father	17,3	NM_001005204.1	missense_variant	293	Q/K	deleterious(0.03)	benign(0.003)		0	0	123	6,047

II.3	PDILT	A>A/C	20371972	16	Snv	PASS	511.77	Father	14,18	NM_174924.1	missense_variant	475	L/R	tolerated(0.31)	benign(0.002)	rs4500734	1.37	2.41	138	6,503

II.6	PDILT	A>A/C	20371972	16	Snv	PASS	283.77	Father	17,12	NM_174924.1	missense_variant	475	L/R	tolerated(0.31)	benign(0.002)	rs4500734	1.37	2.41	138	6,503

II.3	PIH1D1	G>G/A	49949912	19	Snv	PASS	354.77	Father	15,14	NM_017916.2	missense_variant	243	R/C	tolerated(0.07)	possibly_damaging(0.866)	rs149419497	0.41	0.34	61	6,503

II.6	PIH1D1	G>G/A	49949912	19	Snv	PASS	304.77	Father	19,14	NM_017916.2	missense_variant	243	R/C	tolerated(0.07)	possibly_damaging(0.866)	rs149419497	0.41	0.34	61	6,503

II.3	PRX	G>G/A	40900763	19	Snv	PASS	1,056.77	Father	37,36	NM_181882.2	missense_variant	1166	P/S	tolerated(0.55)	benign(0.002)	rs147826200	0	0.07	72	6,503

II.6	PRX	G>G/A	40900763	19	Snv	PASS	1,039.77	Father	30,39	NM_181882.2	missense_variant	1166	P/S	tolerated(0.55)	benign(0.002)	rs147826200	0	0.07	72	6,503

II.3	RYR1	G>G/A	38997024	19	Snv	PASS	717.77	Father	26,25	NM_000540.2	splice_region_variant, intron_variant	0				rs200023171	0.05	0.03	50	6,503

II.6	RYR1	G>G/A	38997024	19	Snv	PASS	529.77	Father	20,18	NM_000540.2	splice_region_variant, intron_variant	0				rs200023171	0.05	0.03	50	6,503

II.3	SHCBP1L	A>A/G	182909488	1	Snv	PASS	928.77	Father	15,30	NM_030933.2	missense_variant	249	I/T	tolerated(0.74)	probably_damaging(0.996)	rs116513797	1.28	0.43	114	6,503

II.6	SHCBP1L	A>A/G	182909488	1	Snv	PASS	798.77	Father	15,26	NM_030933.2	missense_variant	249	I/T	tolerated(0.74)	probably_damaging(0.996)	rs116513797	1.28	0.43	114	6,503

II.3	SIX5	C>C/A	46269196	19	Snv	PASS	1,634.77	Father	57,59	NM_175875.4	missense_variant	595	V/L	tolerated(0.16)	possibly_damaging(0.658)	rs114060947	2.24	0	14	6,478

II.6	SIX5	C>C/A	46269196	19	Snv	PASS	897.77	Father	62,35	NM_175875.4	missense_variant	595	V/L	tolerated(0.16)	possibly_damaging(0.658)	rs114060947	2.24	0	14	6,478

II.3	STK10	G>G/A	171481667	5	Snv	PASS	585.77	Father	33,25	NM_005990.3	missense_variant	853	S/L	tolerated(0.07)	benign(0.146)	rs56066852	0.55	0.99	132	6,503

II.6	STK10	G>G/A	171481667	5	Snv	PASS	419.77	Father	32,19	NM_005990.3	missense_variant	853	S/L	tolerated(0.07)	benign(0.146)	rs56066852	0.55	0.99	132	6,503

II.3	ZNF229	G>G/A	44934187	19	Snv	PASS	974.77	Father	53,39	NM_014518.2	missense_variant	257	R/C	deleterious(0.01)	benign(0.059)	rs144097942	0.55	1.24	106	6,015

II.6	ZNF229	G>G/A	44934187	19	Snv	PASS	534.77	Father	41,23	NM_014518.2	missense_variant	257	R/C	deleterious(0.01)	benign(0.059)	rs144097942	0.55	1.24	106	6,015

II.3	ZNF28	G>G/A	53311347	19	Snv	PASS	896.77	Father	34,33	NM_006969.3	stop_gained	19	Q/*				0	0	0	0

II.6	ZNF28	G>G/A	53311347	19	Snv	PASS	410.77	Father	22,18	NM_006969.3	stop_gained	19	Q/*				0	0	0	0

II.3	ZNF43	T>T/TC	21992330	19	Insertion	PASS	125.77	Father	11,6	NM_001256653.1	frameshift_variant, feature_elongation	179					0	0	0	0

II.6	ZNF43	T>T/TC	21992330	19	Insertion	PASS	185.77	Father	9,7	NM_001256653.1	frameshift_variant, feature_elongation	179					0	0	0	0

II.3	ZNF616	T>T/C	52618555	19	Snv	PASS	581.77	Father	26,20	NM_178523.3	missense_variant	621	N/S	tolerated(0.3)	benign(0.007)	rs116130534	0.05	0	69	6,503

II.6	ZNF616	T>T/C	52618555	19	Snv	PASS	775.77	Father	18,24	NM_178523.3	missense_variant	621	N/S	tolerated(0.3)	benign(0.007)	rs116130534	0.05	0	69	6,503

II.3	ZNF765	T>T/G	53912045	19	Snv	PASS	1,518.77	Father	103,59	NM_001040185.1	missense_variant	413	C/G	deleterious(0)	probably_damaging(0.997)		0	0.02	96	6,503

II.6	ZNF765	T>T/G	53912045	19	Snv	PASS	903.77	Father	47,36	NM_001040185.1	missense_variant	413	C/G	deleterious(0)	probably_damaging(0.997)		0	0.02	96	6,503

II.3	CNTLN	A>C/C	17462985	9	Snv	PASS	1,683.77	Both	0,52	NM_017738.2	missense_variant	1126	E/D	tolerated(0.12)	benign(0.016)	rs142750793	0.37	0.52	84	5,895

II.6	CNTLN	A>C/C	17462985	9	Snv	PASS	981.77	Both	0,32	NM_017738.2	missense_variant	1126	E/D	tolerated(0.12)	benign(0.016)	rs142750793	0.37	0.52	84	5,895

II.3	DPP4	G>C/C	162903930	2	Snv	PASS	1,135.77	Both	0,36	NM_001935.3	missense_variant	59	S/C	deleterious(0.04)	benign(0.249)		0	0	79	6,503

II.6	DPP4	G>C/C	162903930	2	Snv	PASS	880.77	Both	1,26	NM_001935.3	missense_variant	59	S/C	deleterious(0.04)	benign(0.249)		0	0	79	6,503

II.3	KIR2DL1	A>G/G	55285072	19	Snv	PASS	1,063.77	Both	0,33	NM_014218.2	missense_variant	120	I/V	deleterious(0.04)	benign(0.061)	rs138345877	1.42	2.86	197	6,383

II.6	KIR2DL1	A>G/G	55285072	19	Snv	PASS	840.77	Both	0,23	NM_014218.2	missense_variant	120	I/V	deleterious(0.04)	benign(0.061)	rs138345877	1.42	2.86	197	6,383

II.3	LILRB5	G>A/A	54756415	19	Snv	PASS	890.77	Both	0,27	NM_001081442.1	splice_region_variant, intron_variant	0				rs149294774	0.09	0.13	75	6,503

II.6	LILRB5	G>A/A	54756415	19	Snv	PASS	650.77	Both	0,19	NM_001081442.1	splice_region_variant, intron_variant	0				rs149294774	0.09	0.13	75	6,503

II.3	NLRP2	G>A/A	55501424	19	Snv	PASS	1,580.77	Both	2,52	NM_001174081.1	missense_variant	801	A/T	tolerated(0.58)	benign(0.078)	rs117066658	0.78	1.06	113	6,503

II.6	NLRP2	G>A/A	55501424	19	Snv	PASS	1,047.77	Both	0,33	NM_001174081.1	missense_variant	801	A/T	tolerated(0.58)	benign(0.078)	rs117066658	0.78	1.06	113	6,503

II.3	SULT1C2	T>C/C	108921036	2	Snv	PASS	2,210.77	Both	0,65	NM_176825.2	missense_variant	139	Y/H	deleterious(0)	probably_damaging(1)	rs17036091	0.09	0.26	174	6,503

II.6	SULT1C2	T>C/C	108921036	2	Snv	PASS	1,496.77	Both	0,45	NM_176825.2	missense_variant	139	Y/H	deleterious(0)	probably_damaging(1)	rs17036091	0.09	0.26	174	6,503

II.3	TCP10L2	T>C/C	167592524	6	Snv	PASS	1,270.77	Both	0,37	NM_001145121.1	missense_variant	228	L/P	tolerated(0.13)	benign(0)	rs2989545	0	0	24	2,278

II.6	TCP10L2	T>C/C	167592524	6	Snv	PASS	1,366.77	Both	0,38	NM_001145121.1	missense_variant	228	L/P	tolerated(0.13)	benign(0)	rs2989545	0	0	24	2,278

II.3	UBAP2	A>G/G	33944445	9	Snv	PASS	3,558.77	Both	1,110	NM_018449.2	missense_variant	488	I/T	tolerated(0.28)	benign(0.015)	rs201283769	0	0.02	116	6,503

II.6	UBAP2	A>G/G	33944445	9	Snv	PASS	2,596.77	Both	0,75	NM_018449.2	missense_variant	488	I/T	tolerated(0.28)	benign(0.015)	rs201283769	0	0.02	116	6,503

*EVS, Exome databases*.

**Table 4:** Variants inherited from the father alone and not present in other siblings.

Sample	Gene	Variant	Coordinate	Chr	Type	Filters	Quality	Inherited from	Allelic depths	Transcript	Consequence	Protein position	Amino Acids	Sift	PolyPhen	dbSNP ID	Allele Freq Global Minor	Allele Freq EVS	EVS Coverage	EVS Samples
II.3	GXYLT1	C>C/A	42538340	12	Snv	PASS	130.77	Father	3,3	NM_173601.1	missense_variant	37	G/C	tolerated(0.1)	benign(0.186)		0	0	3	4104

II.3	GXYLT1	T>T/C	42538349	12	Snv	PASS	46.77	Father	3,2	NM_173601.1	missense_variant	34	T/A	tolerated(0.78)	benign(0)		0	0	4	4818

II.3	GXYLT1	C>C/A	42538352	12	Snv	PASS	46.77	Father	3,2	NM_173601.1	stop_gained	33	G/*				0	0	4	4931

II.3	GXYLT1	A>A/T	42538366	12	Snv	PASS	46.77	Father	3,2	NM_173601.1	missense_variant	28	V/E	tolerated(0.27)	benign(0.045)		0	0	5	5228

II.3	GXYLT1	C>C/T	42538367	12	Snv	PASS	46.77	Father	3,2	NM_173601.1	missense_variant	28	V/M	tolerated(0.22)	benign(0.161)		0	0	5	5232

II.3	MTFMT	CA>CA/C	65312614	15	Deletion	PASS	35.77	Father	2,3	NM_139242.3	splice_region_variant, intron_variant, feature_truncation	0					0	0	17	5906

II.3	MUC16	A>A/AG	9012894	19	Insertion	PASS	913.77	Father	95,30	NM_024690.2	frameshift_variant, feature_elongation	12,850					0	0	0	0

II.3	MUC16	AG>AG/A	9012897	19	Deletion	PASS	931.77	Father	96,28	NM_024690.2	frameshift_variant, splice_region_variant, feature_truncation	12,849					0	0	211	6170

II.3	PAXBP1	C>C/T	34133365	21	Snv	PASS	322.77	Father	12,13	NM_016631.3	splice_region_variant, intron_variant	0				rs111951332	1.01	1.71	179	6503

II.3	SDHAP1	A>A/G	195690163	3	Snv	PASS	659.77	Father	82,28	NR_003264.2	splice_region_variant, intron_variant, nc_transcript_variant	0				rs201372496	0	0	0	0

II.3	TUBB8	T>T/C	94018	10	Snv	PASS	213.77	Father	86,14	NM_177987.2	missense_variant	105	H/R	deleterious(0.02)	possibly_damaging(0.549)	rs9329307	0	0	68	6503

II.3	URB1	A>A/G	33726265	21	Snv	PASS	1,058.77	Father	67,45	NM_014825.2	missense_variant	798	L/P	tolerated(0.13)	benign(0)	rs189036928	0.92	1.14	116	2283

II.3	URB1	C>C/T	33738971	21	Snv	PASS	704.77	Father	31,28	NM_014825.2	missense_variant	431	V/M	tolerated(0.11)	benign(0.048)	rs117577554	1.14	1.16	92	2283

II.6	AP3B1	T>T/TA	77524068	5	Insertion	PASS	225.77	Father	9,11	NM_003664.3	splice_region_variant, intron_variant, feature_elongation	0				rs35569618, rs5868908	0	0	0	0

II.6	ATP6V1B2	G>G/A	20054928	8	Snv	PASS	438.77	Father	11,20	NM_001693.3	missense_variant	4	R/Q	tolerated(0.49)	benign(0.001)	rs116941637	0.6	0.56	17	6469

II.6	BDP1	A>A/T	70798553	5	Snv	PASS	141.77	Father	8,6	NM_018429.2	missense_variant	726	I/L	tolerated(0.51)	benign(0.002)	rs34588160	0.5	0.48	89	5959

II.6	CAST	C>C/G	96078410	5	Snv	PASS	427.77	Father	32,18	NM_001042440.2	missense_variant	343	R/G	tolerated(0.32)	possibly_damaging(0.66)		0	0	60	6503

II.6	DMGDH	T>T/C	78338202	5	Snv	PASS	882.77	Father	21,35	NM_013391.2	missense_variant	366	N/S	tolerated(0.56)	benign(0.004)	rs77116243	0.92	0.92	120	6503

II.6	FAM182A	C>C/T	26062032	20	Snv	PASS	96.77	Father	24,6	NR_026713.1	splice_region_variant, intron_variant, nc_transcript_variant	0				rs76871018	0	0	7	2190

II.6	gcnt4	G>G/C	74325772	5	Snv	PASS	488.77	Father	21,21	NM_016591.2	missense_variant	31	L/V	tolerated(0.34)	probably_damaging(0.946)		0	0	118	6499

II.6	HOMER1	T>T/A	78692723	5	Snv	PASS	494.77	Father	22,21	NM_004272.3	missense_variant	267	I/L	tolerated(0.84)	benign(0)		0	0	100	5906

II.6	ITPKB	G>G/A	226923779	1	Snv	PASS	899.77	Father	56,40	NM_002221.3	missense_variant	461	P/S	deleterious(0.03)	benign(0.065)	rs35823273	0.18	0.38	35	6501

II.6	NBPF1	T>T/C	16909129	1	Snv	PASS	46.78	Father	1,2	NM_017940.3	missense_variant	406	K/E			rs199798572	0	0	0	0

II.6	NFATC1	G>G/A	77170979	18	Snv	PASS	7,785.77	Father	75,257	NM_172387.1	missense_variant	222	R/Q	deleterious(0.01)	probably_damaging(0.953)		0	0	20	6479

II.6	OBSCN	G>G/A	228462101	1	Snv	PASS	3,090.77	Father	116,114	NM_001098623.1	missense_variant	1,880	C/Y	deleterious(0.02)	probably_damaging(1)		0	0	45	6449

II.6	PCDHA1, PCDHA2, PCDHA3, PCDHA4, PCDHA5, PCDHA6, PCDHA7, PCDHA8	G>G/C	140222138	5	Snv	PASS	1,215.77	Father	84,37	NM_018910.2	intron_variant	0				rs144906391	0	0	119	6492

II.6	PCDHA1, PCDHA2, PCDHA3, PCDHA4, PCDHA5, PCDHA6, PCDHA7, PCDHA8	C>C/CAACT GATCTGATA TATTGTATA GTTTAATA	140222139	5	Insertion	PASS	1,216.77	Father	83,35	NM_018910.2	intron_variant, feature_elongation	0					0	0	0	0

II.6	PCDHA1, PCDHA2, PCDHA3, PCDHA4, PCDHA5, PCDHA6, PCDHA7, PCDHA8	C>C/CTT	140222141	5	Insertion	PASS	1,166.77	Father	83,6	NM_018910.2	intron_variant, feature_elongation	0					0	0	0	0

II.6	PTCD2	C>C/G	71618013	5	Snv	PASS	630.77	Father	29,22	NM_024754.3	missense_variant	48	L/V	deleterious(0.01)	probably_damaging(0.998)		0	0	117	5865

II.6	USH2A	C>C/T	215901623	1	Snv	PASS	445.77	Father	25,17	NM_206933.2	missense_variant	3,939	E/K	tolerated(0.24)	possibly_damaging(0.588)	rs146264950	0.09	0.04	102	6503

*EVS, Exome databases*.

### Genetic Analysis (IV): A Family at Risk of Sudden Death?

**Figure 3:**
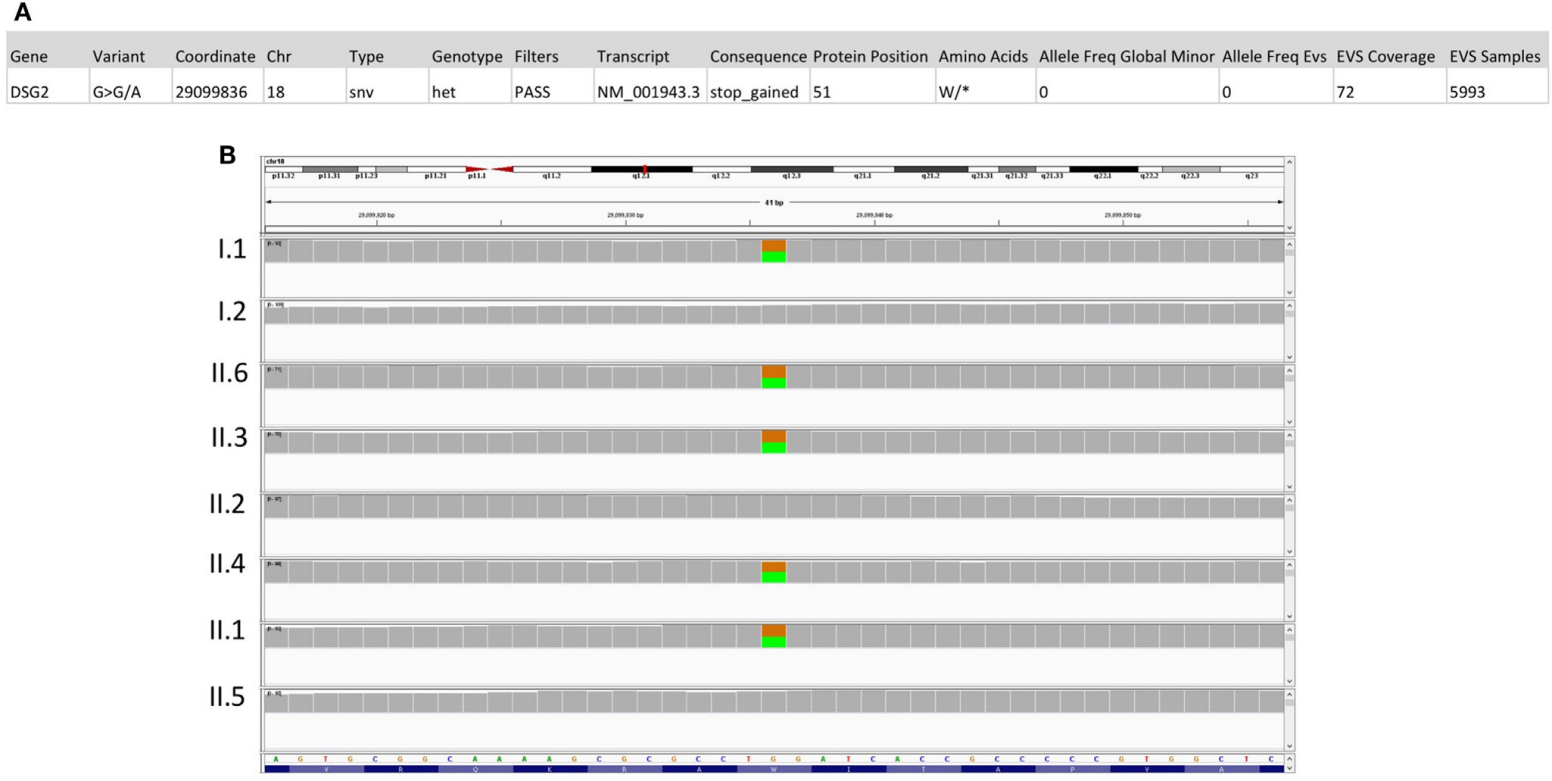
Sequencing results for the *DSG2* variant p.W51*. **(A)** Location and description of the DSG2 variant using the Illumina Variant Studio show that the variant on chr18:290099836 G>A leads to a premature stop codon at position 51 of the transcript with an overall allele frequency of O in the Exome databases (EVS). **(B)** Integrative genomics viewer visualization of whole exome sequencing shows a novel heterozygous variant (brown for the normal “G” allele and green for the variant “A” allele) in *DSG2* in the father (I.1), the three affected children (II.3, II.4, and II.6) and in their healthy sister (II.1).

A final thorough analysis of all predicted deleterious variants in the exome sequencing of patient II.6 showed a novel nonsense mutation in desmoglein (*DSG2*) (Figure 3A), a gene implicated in arrhythmogenic right ventricular dysplasia. The variant is inherited from the father and is shared between four siblings (Figure 3B), including all three affected children with either glaucoma and/or CHD, in addition to a healthy sibling (II.1).

## Discussion

Anterior segment dysgenesis englobes a wild spectrum of ocular defects that include among others the ARS, which is frequently linked to severe functional alterations of either *FOXC1* and/or *PITX2* (28–30). The mutations associated with the ARS phenotype can range from frameshift mutations resulting in premature termination of translation in the forkhead domain or homeodomain, missense mutations reducing transactivation and protein interactions, and nonsense mutations causing haploinsufficiency of the gene product. Most of these mutations will hamper the stability of either one of these transcription factors protein complex over the DNA, and thus have a deleterious effect on transcriptional regulation of target genes (26, 31). Most of the mutations in *FOXC1* appear to be linked to isolated ocular defects or ocular, combined with cardiac, skeletal, and auditory defects. In this study, two of the children who suffered from glaucoma also suffered from CHD. Their mother however, had the same *FOXC1* variant, but did not present with glaucoma or CHD, though she could have defects pertaining to anterior segment dysgenesis. This clinical heterogeneity suggested an important role for modifier factors (genetic, environmental, and/or stochastic) on the phenotypic outcomes. We are thus proposing a digenic model to account for some of the phenotypes in this particular family as deduced from a thorough analysis using WES.

### Ocular Phenotype: Does FOXC1 Need DPT for Glaucoma?

Our results do point out to the determinant role of the p.R127C variant in the ocular phenotype manifested in the three affected children, and in particular to glaucoma which was manifested at very early ages. Only those individuals with this variant do have glaucoma with the exception of the mother, who is a carrier but is glaucoma-free. In search for modifier genes using WES, we could not detect any variation in genes pertaining to the anterior segment dysgenesis phenotype in general and to glaucoma in particular (Figure 1 and Table 1). In parallel, our analysis of the missense variant shows that it is novel and never reported before in the literature. However, the arginine amino acid at position 127 was linked to prior cases of ARS. In particular, a p.R127H variant was associated with an ARS case with the affected proband having severe ocular defects and glaucoma (32). As in our case, this variant was inherited from his mother who does not have glaucoma, but has typical posterior embryotoxon. More recently, a case of ARS was linked to a missense mutation at the same position leading: the p.R127L variant was found in a proband with characteristics of ARS including glaucoma and a cardiac structural defect due to a PDA (33). The patient inherited this variant from his father who was only diagnosed with glaucoma, while the patient’s sister was not available for genetic testing since she passed away as a result of severe dilated cardiomyopathy. As in the published two cases, and in other cases whereby the severity of the ARS phenotype is more pronounced in the children versus their parents (32, 34), we hypothesize that a modifier variant inherited from the *FOXC1* “variant-free parent” would account for the severity of the phenotype in the children versus their parents. Our WES results identified a novel variant in the *DPT* gene inherited from the father and only present in the affected children with glaucoma but not in the healthy children. It could explain the early onset of the anterior segment dysgenesis phenotype and particularly glaucoma in this case. The p.Y149C missense variant is only found in the three affected children with glaucoma: it is predicted to be deleterious and is neither found in healthy Lebanese controls, nor in the gnomAD database (www.gnomad.broadinstitute.org). Additionally, there were no previous reports on the role of *DPT* in glaucoma, and only a few publications describe its expression and role in ocular development and pathology. DPT is an extracellular matrix protein required for the organization of collagen in the skin, as depicted in the mouse knockout model (35). This latter did show also a defect in the corneal matrix organization, which coupled with the prominent expression of the protein in the optic nerve in zebrafish suggest a potential role in ocular development (36, 37). We do suggest a genetic/molecular interaction between *DPT* and *FOXC1*, which would be largely disrupted by the missense mutations detected in our case. A double-heterozygous mouse model carrying only one copy of each gene would potentially yield better insight into this proposed interaction.

### Cardiac Phenotype: A FOXC1/NFATC1 Genetic Interaction

The cardiac phenotype in the indexed-family is divided into two: a mild VSD not requiring any intervention and a severe TOF-like phenotype that required major intervention (Figure 1). We sought that differential variants inherited from the father would contribute to this differential expressivity of the ARS phenotype within the three affected children in this family: two with a cardiac phenotype and the third with only glaucoma. Interestingly, we unravel two novel missense mutations in *OBSCN* (p.C1880Y) and *NFATC1* (p.R222Q) that are predicted to be damaging (Table 4). Both variants are neither present in the Lebanese controls nor in the genome and exome databases. Given that *OBCSN* mutations have not been linked to CHD (38, 39), but could be potentially causing cardiomyopathies, we hypothesize that the *NFATC1* missense variant along the *FOXC1* variant is responsible for the cardiac phenotype. Our rationale is based on previous findings from our group that only a compound mutation in *NFATC1* could be linked to a cardiac phenotype (40), which is also mirrored in the knockout mouse model for *Nfatc1* that shows that the absence of both *Nfatc1* alleles is required to have a severe cardiac phenotype while the heterozygous mice are healthy (41, 42). Both *FOXC1* and *NFATC1* are expressed in the secondary heart field, and thus could be implicated in common transcriptional pathways that shape up the cardiac valves, and septation of the outflow tract (43, 44). This notion of digenic and/or multigenic rationale to explain differential expressivity and penetrance associated with Mendelian-inherited disease is not novel and is being considered in different forms of glaucomas. It was shown that digenic variants in *CYP1B1* and *MYOC* contribute to PCG and that variants in both *FOXC1* and *PITX2* are responsible for some cases of ARS (10, 45). This prompted us to explore the frequency of CHD in patients with ARS carrying a *Foxc1* mutation and whether or not there is a need to carry on WES to investigate the role of other variants in conjunction with *FOXC1* that would explain these cardiac defects.

#### Whole Exome Sequencing

A tool to draw genotype–phenotype correlation out of the 67 *FOXC1* variants reported so far to be linked to the ARS, only nine have been shown to be linked to cardiac defects in addition to the ocular defects. A scrutinized review of the literature of these nine variants, namely p.Q70Hfs*8, p.P79T, p.S82T, p. A85P, p.L86F, p.F112S, p.R127L, p.G149D, and p.R170W, did show that the cardiac phenotype with which they are associated is not as clear as it is presumed. In some cases, the defect is not a structural one, while in most cases, it is found in only one affected child but not in the parent despite sharing the same variant (33, 46). This reinforces the notion that another variant from the healthy parent in a genetic and/or molecular pathway implicating *FOXC1* would be a potential hit to explain the cardiac-associated phenotype. In the case of p.Q70Hfs* 8 and p.P79T, only one affected patient out of two with the variant has an atrial septal defect (ASD) and PDA, respectively, while the parent carrying the mutated allele has a mild ARS phenotype and no cardiac defects (47, 48). The same applies to p.A85P and p.R127L whereby only one of the two affected individuals has either ASD and pulmonary and aortic stenosis or PDA, respectively, whereas the parent from whom the mutation is inherited does not have cardiac defects (33, 49, 50). In the p.S82T case, the initial description of the familial case did not include any structural cardiac defects (17), whereas the report by Mears et al. mentioned cardiac anomalies (14), and that by Du et al. mentioned ASD with neither description of the methods nor the number of affected individuals (33). The same confusion applies to the p.G149D missense mutation, whereby the reported mutation was linked to ASD in one patient from a family whose members were not included, and whereby the information on the cardiac defect was only listed in a table. There was no description of the phenotype in the text, nor in the methodology used to assess it (49). As for the p.F112S missense mutation, the two reports documenting this mutation point out to mitral valve regurgitation and/or congestive heart failure as phenotypes encountered at older ages in only two cases with this genotype whereas the rest did not show any cardiac anomaly (47, 51). The same applies to p.R170W whereby one patient out of five with the mutation has mitral valve regurgitation requiring its replacement, and another an ASD diagnosed in early adolescent (46). Finally, the p.L86F with only one patient having a myocardial infarct at 41 years of age (52). These facts combined with the phenotypes observed in the *Foxc1* initial knockout that did not show any cardiac defects should break down the claims that cardiac structural defects are often associated with the ARS phenotype (24). Indeed, the expression of Foxc1 is barely detected in the mouse developing heart, and only the LacZ harboring knockout construct leads to structural cardiac defects when both *Foxc1* alleles are deleted (47, 53). The deletion of only one allele of *Foxc1* has no effect on heart development in mice even in the context of a genetic model with both *Foxc2* alleles deleted (23, 44). Cardiac defects are thus seldom associated with the ARS phenotype, and we propose that other genes like *NFATC1* when mutated would lead to CHD in the presence of a mutated allele of *FOXC1*.

The limitations in interpreting the cardiac phenotypes in the anterior segment dysgenesis cases could be therefore solved by applying WES for the parents and siblings of any such indexed patient. In our case, the results even go beyond the anterior segment dysgenesis phenotype to highlight a potential life-threat to members of this family with the expression of a nonsense mutation in *DSG2* within its members that could lead to dilated cardiomyopathy and sudden cardiac death.

## Conclusion

This is the first study on a familial case of anterior segment dysgenesis glaucoma in Lebanon, a country with still a high rate of consanguineous marriages. We unravel by WES a novel mutation in *FOXC1* behind the ocular basic phenotype, and we propose a digenic model for the glaucoma phenotype along a mutation in the *DPT* gene and another digenic model for CHD involving yet a novel mutation in *NFATC1*.

## Ethics Statement

All subjects gave written informed consent in accordance with the Declaration of Helsinki. The protocol—Bioch.GN.01—was approved by the Institution Review Board (IRB), at the American University of Beirut.

## Author Contributions

AK: did the experiments, analyzed the data, and wrote the first draft of the paper. CA-H, FB, MK, and MA: did the clinical diagnosis, analyzed the data, and participated in the writing up. HH and KS: did the recruitment and participated in the analysis of the data. GN: conceived the project, supervised the experiments, analyzed the data, participated the writing up, and obtained the funding (with MA).

## Conflict of Interest Statement

The authors declare that the research was conducted in the absence of any commercial or financial relationships that could be construed as a potential conflict of interest.

## References

[1] Leske MC Heijl A Hyman L Bengtsson B Dong L Yang Z Predictors of long-term progression in the early manifest glaucoma trial. Ophthalmology (2007) 114:1965–72. 10.1016/j.ophtha.2007.03.016 17628686

[2] Quigley HA Broman AT. The number of people with glaucoma worldwide in 2010 and 2020. Br J Ophthalmol (2006) 90:262–7. 10.1136/bjo.2005.081224 16488940 PMC1856963

[3] Lewis C Hedberg-Buenz A DeLuca AP Stone EM Alward WLM Fingert JH Primary congenital and developmental glaucomas. Hum Mol Genet (2017) 26(R1):R28–R36. 10.1093/hmg/ddx205 28549150 PMC5886473

[4] Springelkamp H Iglesias AI Mishra A Hohn R Wojciechowski R Khawaja AP New insights into the genetics of primary open-angle glaucoma based on meta-analyses of intraocular pressure and optic disc characteristics. Hum Mol Genet (2017) 26:438–53. 10.1093/hmg/ddw399 28073927 PMC5968632

[5] Liu Y Allingham RR Molecular genetics in glaucoma. Exp Eye Res (2011) 93(4):331–9. 10.1016/j.exer.2011.08.007 21871452 PMC4293633

[6] Rao KN Nagireddy S Chakrabarti S. Complex genetic mechanisms in glaucoma: an overview. Indian J Ophthalmol (2011) 59(Suppl):S31–42. 10.4103/0301-4738.73685 21150032 PMC3038510

[7] Leske MC Wu SY Hennis A Honkanen R Nemesure B Group BES. Risk factors for incident open-angle glaucoma: the Barbados Eye Studies. Ophthalmology (2008) 115:85–93. 10.1016/j.ophtha.2007.03.017 17629563

[8] Leske MC Open-angle glaucoma – an epidemiologic overview. Ophthalmic Epidemiol (2007) 14:166–72. 10.1080/09286580701501931 17896292

[9] Souzeau E Tram KH Witney M Ruddle JB Graham SL Healey PR Myocilin predictive genetic testing for primary open-angle glaucoma leads to early identification of at-risk individuals. Ophthalmology (2017) 124:303–9. 10.1016/j.ophtha.2016.11.011 27993484

[10] Vincent AL Billingsley G Buys Y Levin AV Priston M Trope G Digenic inheritance of early-onset glaucoma: CYP1B1, a potential modifier gene. Am J Hum Genet (2002) 70:448–60. 10.1086/338709 11774072 PMC384919

[11] Zhou T Souzeau E Siggs OM Landers J Mills R Goldberg I Contribution of mutations in known mendelian glaucoma genes to advanced early-onset primary open-angle glaucoma. Invest Ophthalmol Vis Sci (2017) 58:1537–44. 10.1167/iovs.16-21049 28282485

[12] Girgis N Chen TC Genetics of the pediatric glaucomas. Int Ophthalmol Clin (2011) 51:107–17. 10.1097/IIO.0b013e31821e538b 21633242

[13] Ton CC Hirvonen H Miwa H Weil MM Monaghan P Jordan T Positional cloning and characterization of a paired box- and homeobox-containing gene from the aniridia region. Cell (1991) 67:1059–74. 10.1016/0092-8674(91)90284-6 1684738

[14] Mears AJ Jordan T Mirzayans F Dubois S Kume T Parlee M Mutations of the forkhead/winged-helix gene, FKHL7, in patients with Axenfeld-Rieger anomaly. Am J Hum Genet (1998) 63:1316–28. 10.1086/302109 9792859 PMC1377542

[15] Hjalt TA Semina EV. Current molecular understanding of Axenfeld-Rieger syndrome. Expert Rev Mol Med (2005) 7:1–17. 10.1017/S1462399405010082 16274491

[16] Cunningham ET Jr Eliott D Miller NR Maumenee IH Green WR. Familial Axenfeld-Rieger anomaly, atrial septal defect, and sensorineural hearing loss: a possible new genetic syndrome. Arch Ophthalmol (1998) 116:78–82. 10.1001/archopht.116.1.78 9445211

[17] Gould DB Mears AJ Pearce WG Walter MA Autosomal dominant Axenfeld-Rieger anomaly maps to 6p25. Am J Hum Genet (1997) 61:765–8. 10.1016/S0002-9297(07)64340-7 9326342 PMC1715932

[18] Nishimura DY Swiderski RE Alward WL Searby CC Patil SR Bennet SR The forkhead transcription factor gene FKHL7 is responsible for glaucoma phenotypes which map to 6p25. Nat Genet (1998) 19:140–7. 10.1038/493 9620769

[19] Semina EV Reiter R Leysens NJ Alward WL Small KW Datson NA Cloning and characterization of a novel bicoid-related homeobox transcription factor gene, RIEG, involved in Rieger syndrome. Nat Genet (1996) 14:392–9. 10.1038/ng1296-392 8944018

[20] Zhu H. Forkhead box transcription factors in embryonic heart development and congenital heart disease. Life Sci (2016) 144:194–201. 10.1016/j.lfs.2015.12.001 26656470

[21] Golson ML Kaestner KH. Fox transcription factors: from development to disease. Development (2016) 143:4558–70. 10.1242/dev.112672 27965437 PMC5201025

[22] Lehmann OJ Sowden JC Carlsson P Jordan T Bhattacharya SS Fox’s in development and disease. Trends Genet (2003) 19:339–44. 10.1016/S0168-9525(03)00111-2 12801727

[23] Kume T Jiang H Topczewska JM Hogan BL. The murine winged helix transcription factors, Foxc1 and Foxc2, are both required for cardiovascular development and somitogenesis. Genes Dev (2001) 15:2470–82. 10.1101/gad.907301 11562355 PMC312788

[24] Kume T Deng KY Winfrey V Gould DB Walter MA Hogan BL. The forkhead/winged helix gene Mf1 is disrupted in the pleiotropic mouse mutation congenital hydrocephalus. Cell (1998) 93:985–96. 10.1016/S0092-8674(00)81204-0 9635428

[25] Smith RS Zabaleta A Kume T Savinova OV Kidson SH Martin JE Haploinsufficiency of the transcription factors FOXC1 and FOXC2 results in aberrant ocular development. Hum Mol Genet (2000) 9:1021–32. 10.1093/hmg/9.7.1021 10767326

[26] Ito YA Goping IS Berry F Walter MA. Dysfunction of the stress-responsive FOXC1 transcription factor contributes to the earlier-onset glaucoma observed in Axenfeld-Rieger syndrome patients. Cell Death Dis (2014) 5:e1069. 10.1038/cddis.2014.8 24556684 PMC3944279

[27] Al-Haddad C Abdulaal M Badra R Barikian A Noureddine B Farra C. Genotype/phenotype correlation in primary congenital glaucoma patients in the lebanese population: a pilot study. Ophthalmic Genet (2016) 37:31–6. 10.3109/13816810.2014.924015 24940937

[28] Souzeau E Siggs OM Zhou T Galanopoulos A Hodson T Taranath D Glaucoma spectrum and age-related prevalence of individuals with FOXC1 and PITX2 variants. Eur J Hum Genet (2017) 25:839–47. 10.1038/ejhg.2017.59 28513611 PMC5520071

[29] Tumer Z Bach-Holm D. Axenfeld-Rieger syndrome and spectrum of PITX2 and FOXC1 mutations. Eur J Hum Genet (2009) 17:1527–39. 10.1038/ejhg.2009.93 19513095 PMC2987033

[30] Reis LM Tyler RC Volkmann Kloss BA Schilter KF Levin AV Lowry RB PITX2 and FOXC1 spectrum of mutations in ocular syndromes. Eur J Hum Genet (2012) 20:1224–33. 10.1038/ejhg.2012.80 22569110 PMC3499749

[31] Komatireddy S Chakrabarti S Mandal AK Reddy AB Sampath S Panicker SG Mutation spectrum of FOXC1 and clinical genetic heterogeneity of Axenfeld-Rieger anomaly in India. Mol Vis (2003) 9:43–8. 12592227

[32] Kawase C Kawase K Taniguchi T Sugiyama K Yamamoto T Kitazawa Y Screening for mutations of Axenfeld-Rieger syndrome caused by FOXC1 gene in Japanese patients. J Glaucoma (2001) 10:477–82. 10.1097/00061198-200112000-00007 11740218

[33] Du RF Huang H Fan LL Li XP Xia K Xiang R A novel mutation of FOXC1 (R127L) in an axenfeld-rieger syndrome family with glaucoma and multiple congenital heart diseases. Ophthalmic Genet (2016) 37:111–5. 10.3109/13816810.2014.924016 24914578

[34] Khan AO Aldahmesh MA Al-Amri A. Heterozygous FOXC1 mutation (M161K) associated with congenital glaucoma and aniridia in an infant and a milder phenotype in her mother. Ophthalmic Genet (2008) 29:67–71. 10.1080/13816810801908152 18484311

[35] Takeda U Utani A Wu J Adachi E Koseki H Taniguchi M Targeted disruption of dermatopontin causes abnormal collagen fibrillogenesis. J Invest Dermatol (2002) 119:678–83. 10.1046/j.1523-1747.2002.01863.x 12230512

[36] Tan Y Iimura K Sato T Ura K Takagi Y. Spatiotemporal expression of the dermatopontin gene in zebrafish *Danio rerio*. Gene (2013) 516:277–84. 10.1016/j.gene.2012.11.074 23266816

[37] Cooper LJ Bentley AJ Nieduszynski IA Talabani S Thomson A Utani A The role of dermatopontin in the stromal organization of the cornea. Invest Ophthalmol Vis Sci (2006) 47:3303–10. 10.1167/iovs.05-1426 16877395 PMC1868961

[38] Marston S. Obscurin variants and inherited cardiomyopathies. Biophys Rev (2017) 9(3):239–43. 10.1007/s12551-017-0264-8 28510120 PMC5498328

[39] Marston S Montgiraud C Munster AB Copeland O Choi O Dos Remedios C OBSCN mutations associated with dilated cardiomyopathy and haploinsufficiency. PLoS One (2015) 10:e0138568. 10.1371/journal.pone.0138568 26406308 PMC4583186

[40] Abdul-Sater Z Yehya A Beresian J Salem E Kamar A Baydoun S Two heterozygous mutations in NFATC1 in a patient with tricuspid atresia. PLoS One (2012) 7:e49532. 10.1371/journal.pone.0049532 23226213 PMC3511479

[41] Ranger AM Grusby MJ Hodge MR Gravallese EM de la Brousse FC Hoey T The transcription factor NF-ATc is essential for cardiac valve formation. Nature (1998) 392:186–90. 10.1038/32426 9515964

[42] de la Pompa JL Timmerman LA Takimoto H Yoshida H Elia AJ Samper E Role of the NF-ATc transcription factor in morphogenesis of cardiac valves and septum. Nature (1998) 392:182–6. 10.1038/32419 9515963

[43] Lin CY Lin CJ Chen CH Chen RM Zhou B Chang CP. The secondary heart field is a new site of calcineurin/Nfatc1 signaling for semilunar valve development. J Mol Cell Cardiol (2012) 52:1096–102. 10.1016/j.yjmcc.2012.01.013 22300732 PMC3327781

[44] Seo S Kume T. Forkhead transcription factors, Foxc1 and Foxc2, are required for the morphogenesis of the cardiac outflow tract. Dev Biol (2006) 296:421–36. 10.1016/j.ydbio.2006.06.012 16839542

[45] Kelberman D Islam L Holder SE Jacques TS Calvas P Hennekam RC Digenic inheritance of mutations in FOXC1 and PITX2: correlating transcription factor function and Axenfeld-Rieger disease severity. Hum Mutat (2011) 32:1144–52. 10.1002/humu.21550 21837767

[46] Gripp KW Hopkins E Jenny K Thacker D Salvin J. Cardiac anomalies in Axenfeld-Rieger syndrome due to a novel FOXC1 mutation. Am J Med Genet A (2013) 161A:114–9. 10.1002/ajmg.a.35697 23239455

[47] Swiderski RE Reiter RS Nishimura DY Alward WL Kalenak JW Searby CS Expression of the Mf1 gene in developing mouse hearts: implication in the development of human congenital heart defects. Dev Dyn (1999) 216:16–27. 10.1002/(SICI)1097-0177(199909)216:1<16::AID-DVDY4>3.0.CO;2-1 10474162

[48] Suzuki T Takahashi K Kuwahara S Wada Y Abe T Tamai M A novel (Pro79Thr) mutation in the FKHL7 gene in a Japanese family with Axenfeld-Rieger syndrome. Am J Ophthalmol (2001) 132:572–5. 10.1016/S0002-9394(01)01059-5 11589884

[49] Weisschuh N Dressler P Schuettauf F Wolf C Wissinger B Gramer E. Novel mutations of FOXC1 and PITX2 in patients with Axenfeld-Rieger malformations. Invest Ophthalmol Vis Sci (2006) 47:3846–52. 10.1167/iovs.06-0343 16936096

[50] Fuse N Takahashi K Yokokura S Nishida K. Novel mutations in the FOXC1 gene in Japanese patients with Axenfeld-Rieger syndrome. Mol Vis (2007) 13:1005–9. 17653043 PMC2776537

[51] Honkanen RA Nishimura DY Swiderski RE Bennett SR Hong S Kwon YH A family with Axenfeld-Rieger syndrome and Peters Anomaly caused by a point mutation (Phe112Ser) in the FOXC1 gene. Am J Ophthalmol (2003) 135:368–75. 10.1016/S0002-9394(02)02061-5 12614756

[52] Saleem RA Banerjee-Basu S Berry FB Baxevanis AD Walter MA. Structural and functional analyses of disease-causing missense mutations in the forkhead domain of FOXC1. Hum Mol Genet (2003) 12:2993–3005. 10.1093/hmg/ddg324 14506133

[53] Winnier GE Kume T Deng K Rogers R Bundy J Raines C Roles for the winged helix transcription factors MF1 and MFH1 in cardiovascular development revealed by nonallelic noncomplementation of null alleles. Dev Biol (1999) 213:418–31. 10.1006/dbio.1999.9382 10479458

