# Supplementary material for: A Novel Mutation in FOXC1 in a Lebanese Family with Congenital Heart Disease and Anterior Segment Dysgenesis: Potential Roles for NFATC1 and DPT in the Phenotypic Variations
Source: Front Cardiovasc Med. 2017 Sep 20;4:58. doi: 10.3389/fcvm.2017.00058 (PMC5611365; doi:10.3389/fcvm.2017.00058)
Supplement: Supplementary file 1 [file table_1.doc]

**Supplementary Table 1:** Genes linked to anterior segment dysgenesis, microcornea, and microphthalmia

| Gene Name |
| --- |
| ADAMTS18 |
| ALDH1A3 |
| ATOH7 |
| B3GATLTL |
| BCOR |
| BEST1 |
| BMP4 |
| CAV1 |
| CAV2 |
| CDKN2B |
| CNTNAP2 |
| COL11A1 |
| COL4A1 |
| CRYAA |
| CRYBA4 |
| CRYBB1 |
| CRYBB2 |
| CRYGC |
| CRYGD |
| CTDP1 |
| CYP1B1 |
| EGR1 |
| EYA1 |
| FOXC1 |
| FOXC2 |
| FOXE3 |
| GALC |
| GDF6 |
| GJA8 |
| HCCS |
| LMX1B |
| LOXL1 |
| LRP12 |
| LTBP2 |
| MACOM |
| MAF |
| MFRP |
| MIR184 |
| MYOC |
| NHS |
| ODZ3 |
| OPTN |
| OTX2 |
| PAX6 |
| PCMTD1 |
| PITX2 |
| PITX3 |
| PLEKHA7 |
| PRSS56 |
| PXDN |
| RAB18 |
| RAB3GAP1 |
| RAB3GAP2 |
| RAX |
| SHH |
| SIX1 |
| SIX6 |
| SLC16A12 |
| SOX2 |
| ST18 |
| STRA6 |
| TBK1 |
| TMCO1 |
| VSX2 |
| WDR36 |
| ZFPM2 |
